# Disentangling the Impact of the COVID‐19 Lockdowns on Urban NO_2_ From Natural Variability

**DOI:** 10.1029/2020GL089269

**Published:** 2020-09-05

**Authors:** Daniel L. Goldberg, Susan C. Anenberg, Debora Griffin, Chris A. McLinden, Zifeng Lu, David G. Streets

**Affiliations:** ^1^ Department of Environmental and Occupational Health George Washington University Washington DC USA; ^2^ Energy Systems Division Argonne National Laboratory Lemont IL USA; ^3^ Air Quality Research Division Environment and Climate Change Canada (ECCC) Toronto Ontario Canada

**Keywords:** COVID‐19, NO_*x*_ emissions, TROPOMI NO_2_, meteorological effects, NO_2_ trends

## Abstract

TROPOMI satellite data show substantial drops in nitrogen dioxide (NO_2_) during COVID‐19 physical distancing. To attribute NO_2_ changes to NO_*x*_ emissions changes over short timescales, one must account for meteorology. We find that meteorological patterns were especially favorable for low NO_2_ in much of the United States in spring 2020, complicating comparisons with spring 2019. Meteorological variations between years can cause column NO_2_ differences of ~15% over monthly timescales. After accounting for solar angle and meteorological considerations, we calculate that NO_2_ drops ranged between 9.2% and 43.4% among 20 cities in North America, with a median of 21.6%. Of the studied cities, largest NO_2_ drops (>30%) were in San Jose, Los Angeles, and Toronto, and smallest drops (<12%) were in Miami, Minneapolis, and Dallas. These normalized NO_2_ changes can be used to highlight locations with greater activity changes and better understand the sources contributing to adverse air quality in each city.

## Key Points

- Meteorological patterns were especially favorable for low NO_2_ in much of the United States in spring 2020, complicating comparisons with spring 2019
- Weather variations between years can cause column NO_2_ differences of ~15% over monthly timescales
- NO_2_ drops attributed to COVID‐19 lockdowns ranged between 9.2% and 43.4% among 20 cities in North America, with a median of 21.6%

## 1 Introduction

Nitrogen dioxide (NO_2_) is unique due to its relatively short photochemical lifetime, which varies from 2–6 hr during the summer daytime (Beirle et al., 2011; de Foy et al., 2014; Laughner & Cohen, 2019; Valin et al., 2013) to 12–24 h during winter (Beirle et al., 2003; Shah et al., 2020); the main loss pathway of NO_2_ is reaction with OH (Stavrakou et al., 2013). Due to the relatively short lifetime of NO_2_, tropospheric NO_2_ concentrations are strongly correlated with local NO_*x*_ emissions, which are often anthropogenic in origin. However, due to the effects of meteorology and solar zenith angle on the NO_2_ abundance, NO_2_ can vary by a factor of two simply due to seasonal changes (Pope et al., 2015; Wang et al., 2019). Therefore, satellite data are typically averaged over long timeframes (approximately seasonal/annual) to assess changes in NO_*x*_ emissions (Duncan et al., 2016; Geddes et al., 2016; Georgoulias et al., 2019; Hilboll et al., 2013, 2017; Kim et al., 2009; Krotkov et al., 2016; Lamsal et al., 2015; McLinden et al., 2016; van Der et al., 2008).

With the COVID‐19 crisis, there is now broad interest in rapid assessments of NO_*x*_ emission changes on short timescales in locations that have implemented stay‐at‐home orders or other physical distancing measures. Using satellite data in this instance can be advantageous due to its global coverage at immediate timescales. However, current methods of averaging satellite NO_2_ data over many months to minimize random daily effects of weather will not provide the temporal granularity needed to quantify short‐lived NO_*x*_ emission changes.

Preliminary satellite‐based studies indicate that NO_2_ dropped substantially in China following stringent COVID‐19 physical distancing actions (Liu et al., 2020; Zhang et al., 2020). Similar declines have also been seen over northern Italy (ESA, 2020b) and India (ESA, 2020a). Although lockdown measures—and adherence to them—have been looser in the United States than in China, India, and Italy, preliminary analyses show that NO_2_ amounts are declining across United States cities as well (NASA, 2020). These declines have, in some cases in the media (Holcombe & O'Key, 2020; Plumer & Popovich, 2020), been attributed to the emission changes during lockdowns, without accounting for the potentially substantial influences of meteorology and seasonality. Accounting for natural NO_2_ fluctuations are especially important during spring, a time when the NO_2_ concentrations and lifetimes are quickly changing due to transitioning meteorology, solar zenith angle, and snow cover.

Understanding how NO_*x*_ emissions have changed in response to physical distancing measures requires new methods to account for solar zenith angle and meteorological conditions over very short time scales (days/weeks), as opposed to the traditional method of averaging over seasons and years. Here, we use three different methods to assess the NO_2_ decreases associated with COVID‐19 lockdowns. We combine TROPOMI NO_2_ data with ERA5 reanalysis and a regional chemical transport model to determine the effects of the solar zenith angle and meteorological factors—such as wind speed and wind direction—on NO_2_ column amounts. The NO_2_ changes after this “normalization” are more likely to represent the NO_*x*_ emissions changes due to COVID‐19.

## 2 Methods

### 2.1 TROPOMI NO_2_

TROPOMI was launched by the European Space Agency (ESA) for the European Union's Copernicus Sentinel 5 Precursor (S5p) satellite mission on 13 October 2017. The satellite follows a Sun‐synchronous, low‐Earth (825 km) orbit with a daily equator overpass time of approximately 13:30 local solar time (van Geffen et al., 2019). TROPOMI measures total column amounts of several trace gases in the Ultraviolet‐Visible‐Near Infrared‐Shortwave Infrared spectral regions (Veefkind et al., 2012). At nadir, pixel sizes are 3.5 × 7 km^2^ (reduced to 3.5 × 5.6 km^2^ on 6 August 2019) with little variation in pixel sizes across the 2,600 km swath.

Using a differential optical absorption spectroscopy (DOAS) technique on the radiance measurements in the 405–465 nm spectral window, the top‐of‐atmosphere spectral radiances can be converted into slant column amounts of NO_2_ between the sensor and the Earth's surface (Boersma et al., 2018). In two additional steps, the slant column quantity can be converted into a tropospheric vertical column content, which is the quantity used most often to further our understanding of NO_2_ in the atmosphere (Beirle et al., 2019; Dix et al., 2020; Goldberg, Lu, Streets, et al., 2019; Griffin et al., 2019; Ialongo et al., 2020; Reuter et al., 2019; Zhao et al., 2020). For this analysis, we use the operational “off‐line” TROPOMI NO_2_ data set, Version 1.2 preceding 26 March 2019 and Version 1.3 post‐27 March 2019.

### 2.2 Meteorological Data Set

We use ERA5 meteorology (Copernicus Climate Change Service (C3S), 2017) for the wind speed and direction in our analysis. When filtering the data based on wind, we use the average 100‐m winds during 16–21 UTC, which approximately corresponds to the TROPOMI overpass time over North America. To downscale the ERA5 reanalysis, which is provided at 0.25° × 0.25°, we spatially interpolate daily averaged winds to 0.01° × 0.01° using bilinear interpolation. Due to our dependence on 0.25° × 0.25° meteorology, any microscale features (e.g., sea breezes) will not be accounted for, but these effects should be minor for our particular analysis.

### 2.3 Calculation of NO_2_ Changes

We calculate the NO_2_ changes using three different methods and a control. In the control (Method 0), a simple difference before (1 January to 29 February 2020) and after (15 March to 30 April 2020) COVID‐19 precautions is calculated; this represents the true change in NO_2_ column densities over time. The 15 March to 30 April 2020 period is the timeframe of the most stringent lockdown in North America—some U.S. states began to transition out of the lockdown on 1 May 2020. In Method 1, we compare an average of 15 March to 30 April 2020 to the same timeframe of 2019 and would therefore account for impact of changes due to solar zenith angle; this year‐over‐year comparison is used most often in satellite studies quantifying long‐term changes in NO_*x*_ emissions. In Method 2, we develop a strategy to account for varying weather patterns without the use of a chemical transport model. In this method, we normalize each day's NO_2_ observation to a day with “standard” meteorology—similar to standard temperature and pressure (STP) conditions in a laboratory setting. We do this by accounting for four different day‐varying effects; these are solar zenith angle, wind speed, wind direction, and day‐of‐week. In all cases, we normalize city‐specific conditions to those that are climatological on 15 April. Finally, in Method 3, we infer a TROPOMI NO_2_ column amount assuming no COVID‐19 precautions using the GEM‐MACH regional chemical transport model, which is operationally run in forecast mode (Pendlebury et al., 2018), and then compare the actual TROPOMI columns to the theoretical columns. Methods 2 and 3 both account for year‐varying meteorology, while Method 1 does not. A detailed description of Methods 2 and 3 can be found in the supporting information.

## 3 Results

### 3.1 Solar Zenith Angle and Meteorological Relationships

**Figure 1:**
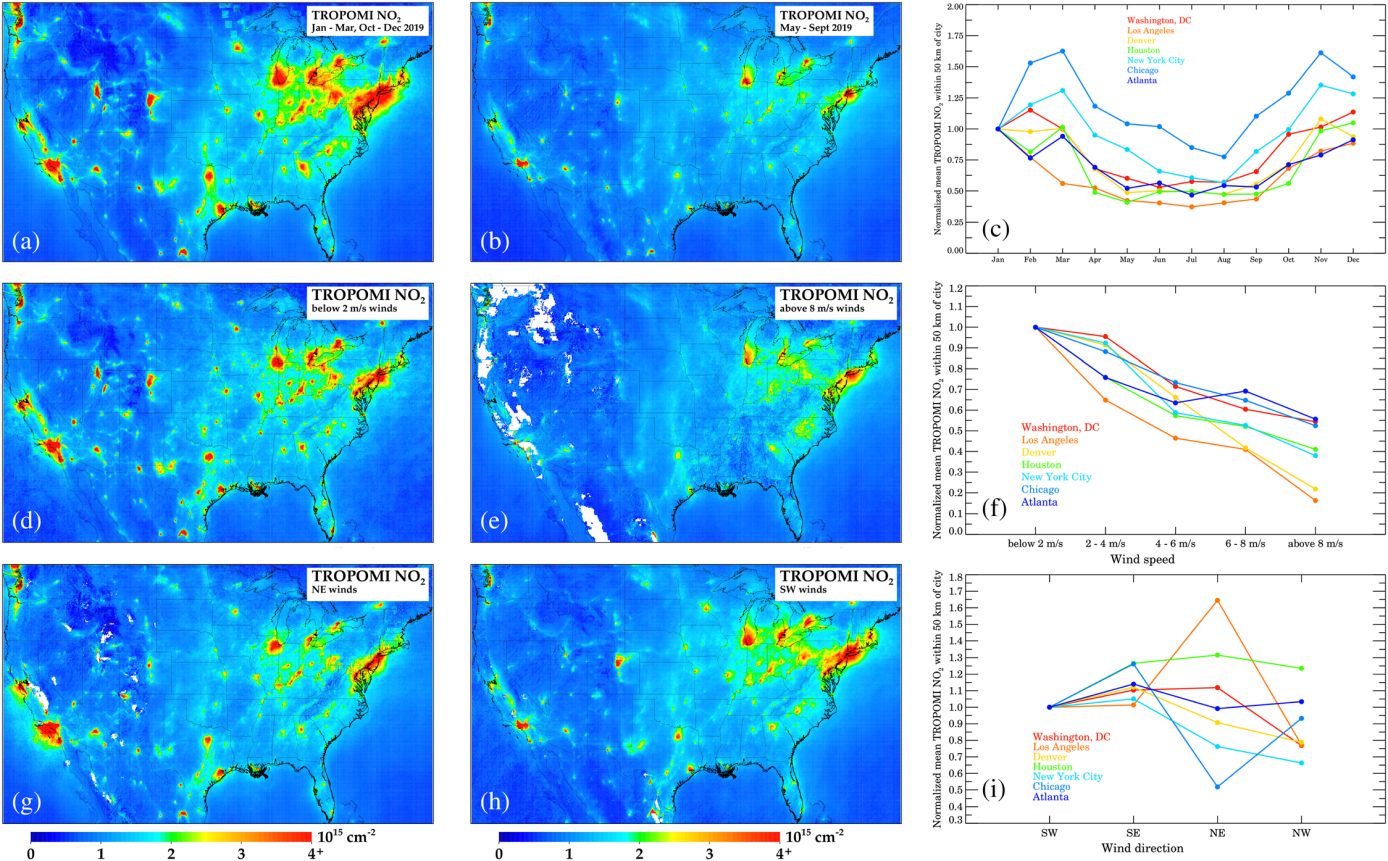
Effects of meteorology and solar zenith angle on column NO_2_. Top panels show (a) TROPOMI NO_2_ during the warm season (May–Sept 2019), (b) during the cold season (January–March and October–December 2019), and (c) the monthly variation in seven U.S. cities normalized to January 2019. Middle panels show (d) TROPOMI NO_2_ when winds are <2 m/s, (e) when winds are >8 m/s, and (f) variations in NO_2_ as a function of wind speed for seven cities normalized to stagnant conditions. Bottom panels show (g) TROPOMI NO_2_ when winds are northeasterly, (h) when winds are southwesterly, and (i) variations as a function of wind direction for seven cities normalized to southwesterly winds. Wind variations are using the complete TROPOMI record preceding 1 January 2020 (1 May 2018 to 31 December 2019).

In the top row of Figure 1, we show 2019 NO_2_ column densities during the high solar zenith angle “cold” season (January–March and October–December) and low solar zenith angle “warm” season (May–September) in the continental United States and southern Canada.

Column NO_2_ is larger during the cold season than during the warm season over the majority of our domain, despite NO_*x*_ emissions generally peaking during summer months due to a heavy air conditioning load (Abel et al., 2017; He et al., 2013) and more vehicle miles traveled (https://www.transtats.bts.gov/osea/seasonaladjustment/?PageVar=VMT). The larger NO_2_ concentrations during the winter are instead due to the longer NO_2_ lifetime during the cold season, primarily due to slower photolysis rates. When NO_*x*_ is emitted during the warm season, it is transformed into other chemical species, such as O_3_ and HNO_3_, more quickly than during the winter. Also, the fraction of NO_*x*_ that is in the form of NO_2_ (rather than NO) is variable, largely due to relative levels of O_3_ and sunlight. We find that in most near‐urban locations column NO_2_ amounts are 1.5–3 times larger during the winter than during the summer and can vary substantially between city.

In a next step, we account for wind speed and wind direction in the spatiotemporal variation of NO_2_ columns. In the middle and bottom panels of Figure 1, we demonstrate the effects of wind speed and wind direction on the NO_2_ in our domain. Increases in wind speed yield NO_2_ decreases due to quicker dispersion away from the city centers. For example, in New York City, Washington DC, Atlanta, and Chicago, all cities with relatively flat topography and located in the eastern United States, increasing wind speeds from nearly stagnant to >8 m/s decreases NO_2_ by 30–60%. Conversely, in Denver and Los Angeles, cities with more heterogeneous topography and with general isolation from an agglomeration of cities show a stronger dependence on wind speed; increasing wind speeds from nearly stagnant to >8 m/s decreases NO_2_ by 70–85%. In both instances, these examples show the strong dependence of wind speed on local NO_2_ amounts.

Similarly, wind direction has a large role in the local NO_2_ amounts, although the effects of wind direction are nonlinear. Generally, northwest winds yield the cleanest conditions in most U.S. cities, but the effects of other wind directions are more nuanced. For example, southwesterly winds yield the worst air quality in New York City, while northeasterly winds yield the largest NO_2_ in Washington, D.C. This is due to the fact that the other city lies upwind in each opposing scenario. Changes in wind direction, given the same wind speed, can yield differences in NO_2_ in major cities by up to 70%, and must be accounted for if properly attributing NO_2_ changes to NO_*x*_ emissions. Climatological patterns for all cities are shown in the supporting information (Figures S2–S4).

While 2‐m air temperature and boundary layer depth may be affecting the NO_2_ concentrations, these are not independent of the aforementioned factors: solar zenith angle, wind speed, and wind direction. In fact, solar zenith angle, wind speed, and wind direction are by themselves highly skilled predictors of near‐surface temperatures and boundary layer depth in most instances. Since we are focused on mostly clear‐sky days, clouds have limited effects here. Previous day's precipitation may also be a contributing factor to daily NO_2_ amounts, but in many areas, the wind direction will partially account for this, since northwest winds usually follow large rain events in most areas.

### 3.2 Effects of COVID‐19 Physical Distancing on NO_2_

In order to quantify rapid changes in NO_*x*_ due to COVID‐19 physical distancing, we calculate NO_2_ changes in North American cities using three different methods and a reference method. The results for all cities are shown in Table 1.

**Table 1:** Percentage Drop in Column NO_2_ as Observed by TROPOMI

	Reference case	Account for solar zenith angle only	Account for solar zenith angle and meteorology			
	Method 0	Method 1	Method 2	Method 3	Mean of methods 1–3	Median of methods 1–3
City name	Δ between months 2020 only (January–February vs. 15 March to 30 April)	Δ between years 2019 vs. 2020 (15 March to 30 April)	Using ERA5 analogs to account for meteorology 2019 versus 2020 (15 March to 30 April)	Using GEM‐MACH to infer NO_2_, 2020 only (15 March to 30 April)		
San Jose	65.2%	43.4%	40.7%	43.5%	42.5%	**43.4%**
Los Angeles	66.1%	32.6%	32.5%	38.6%	34.6%	**32.6%**
Toronto	60.4%	31.0%	17.0%	42.0%	30.0%	**31.0%**
Philadelphia	50.3%	36.6%	30.7%	22.1%	29.8%	**30.7%**
Denver	25.8%	29.2%	23.4%	39.1%	30.6%	**29.2%**
Atlanta	39.6%	35.2%	27.4%	20.2%	27.6%	**27.4%**
Detroit	35.5%	29.9%	22.8%	15.6%	22.8%	**22.8%**
Boston	40.3%	22.8%	23.5%	17.8%	21.4%	**22.8%**
Washington DC	42.9%	31.4%	21.2%	6.7%	19.8%	**21.2%**
Montreal	12.5%	3.3%	20.9%	30.2%	18.1%	**20.9%**
New York City	32.7%	20.2%	20.0%	17.9%	19.4%	**20.0%**
New Orleans	41.7%	13.5%	19.6%	22.5%	18.5%	**19.6%**
Las Vegas	66.7%	9.5%	18.4%	42.0%	23.3%	**18.4%**
Houston	38.9%	26.3%	15.6%	1.9%	14.6%	**15.6%**
Chicago	31.0%	23.6%	14.9%	3.5%	14.0%	**14.9%**
Phoenix	43.9%	12.8%	14.8%	35.4%	21.0%	**14.8%**
Austin	34.3%	14.5%	9.4%	16.1%	13.3%	**14.5%**
Dallas	41.9%	11.9%	3.6%	16.7%	10.7%	**11.9%**
Miami	27.9%	16.1%	−1.6%	11.0%	8.5%	**11.0%**
Minneapolis	0.1%	14.3%	9.2%	8.1%	10.5%	**9.2%**
Mean of each method	39.9%	22.9%	19.2%	22.5%	21.6%	**21.6%**

The reference method, Method 0, compares the prelockdown and postlockdown periods and represents the “true” NO_2_ change; however, this method does not account for seasonal changes and, thus, is not considered in the medians/means.

**Figure 2:**
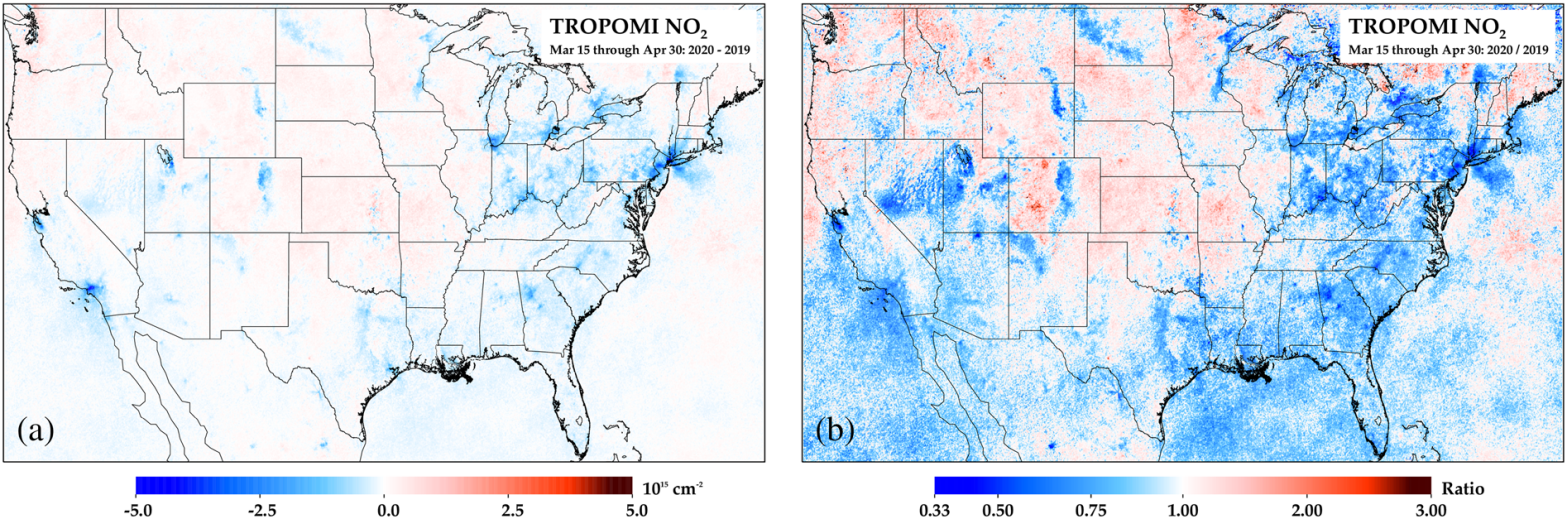
TROPOMI NO_2_ differences between 2019 and 2020, using 15 March to 30 April 2020 as the post‐COVID‐19 period. Plots are showing (a) the absolute difference and (b) the ratio between years.

In Method 1, we compare an average of 15 March to 30 April 2020 to the same timeframe of 2019. In Figure 2, we show difference and ratio plots between these 2 years (i.e., Method 1). The largest decreases in NO_2_ are near the major cities in North America. We also find regional decreases in the eastern North America. Conversely, the central and northwestern United States have seen little change between years, which is likely due to the high fraction of NO_2_ attributed to biogenic sources and long‐range transport. We also observe substantial decreases near retired electricity generating units in the western United States (Storrow, 2019).

**Figure 3:**
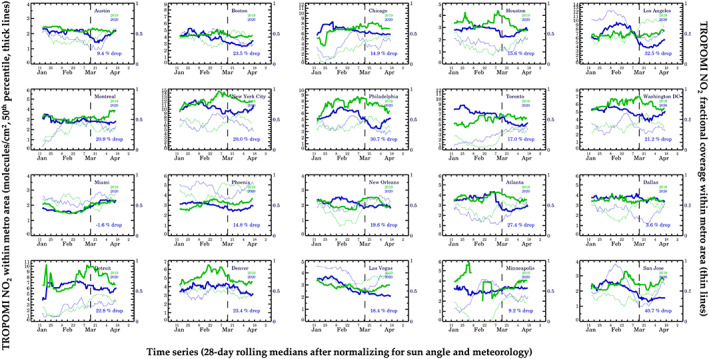
Trends in TROPOMI NO_2_ since 1 January in 2019 and 2020 after accounting for meteorological variability and solar zenith angle. The thick lines represent the 28‐day rolling median value (50th percentile) in a 0.4° × 0.4° box centered on the city center for the largest cities (New York City, Los Angeles, Chicago, Toronto, and Houston) and 0.2° × 0.2° box in all other cities. The thin lines represent the fractional coverage (0–1) in the coincident spatiotemporal domain.

In Figure 3, we demonstrate Method 2. Here, we show the 2019 and 2020 28‐day running TROPOMI NO_2_ medians after accounting for solar zenith angle, day‐of‐week patterns, and two meteorological factors: wind speed and wind direction. A 28‐day period is chosen so as to best average out any random fluctuations not associated with meteorological influences, such as missing data or real changes in NO_*x*_ emissions due to temperature changes, but there may have been some fluctuations that cannot be averaged out. In Figure 3, the January values are uniformly lower than their true values (Figure S5) because we are normalizing to April meteorological conditions (i.e., solar zenith angle is lower in April as compared to January). In New York City, we calculate a 20.0% drop in NO_2_ due to COVID‐19 precautions. We find that there is no difference between Method 2—which accounts for meteorology—and Method 1—which only accounts for solar zenith angle. This suggests that varying meteorological conditions in New York City, while different between years, may not have had a strong biasing effect. However, in Washington D.C., we find favorable conditions in 2020 as compared to 2019 because we observe substantially different NO_2_ drops before (31.4%) and after (21.2%) correcting for the meteorology. These results are corroborated by the wind speed and direction (Figure S6). In 2019, winds were on average southwesterly, while in 2020, winds had more of a northwesterly and therefore cleaner component. Of all cities analyzed, we find that Miami had the most favorable conditions for low NO_2_ in 2020 as compared to 2019; in 2020, winds were stronger from the south—in this case a cleaner air mass—than in 2019, which had relatively stagnant winds. Conversely, in Montreal, New Orleans, and Las Vegas, meteorological conditions appeared to be unfavorable in 2020 as compared to 2019.

Meteorological factors that we do not account for in Method 2 are a difference in the number of cloudy scenes and the amount of snow cover between years. In particular, the northern United States had more snow cover in late February and early March in 2019 than in 2020 (Figure S7). In Figure 3, we also show the fractional coverage of the metropolitan area during the 28‐day period. For example, in New York City, there was ~0.6 fractional coverage in early March 2020, while in 2019 there was only ~0.3 fractional coverage during the same timeframe. As a result, some of the higher values during this timeframe may be related to fewer valid pixels in outlying areas, which retain snow for longer, and perhaps a snow‐related reflectivity artifact even though pixels over snow cover are predominantly removed. This is also why some other snow‐prone cities like Chicago, Philadelphia, Toronto, Detroit, Denver, and Minneapolis show large variability preceding March. Beginning in mid‐March, snow cover was largely gone in most U.S. cities during both years.

**Figure 4:**
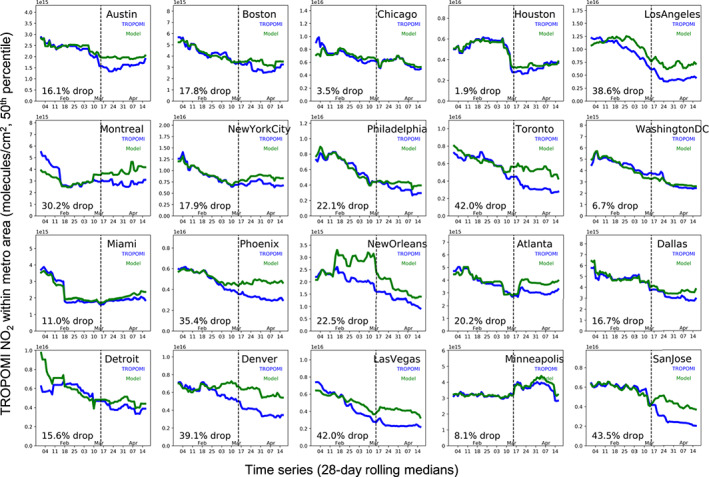
Trends in TROPOMI NO_2_ since 1 January 2020. The actual observed columns are shown in black, while the “expected” columns—using GEM‐MACH to infer NO_2_ in the absence of lockdowns—is shown in blue. The lines represent the 28‐day rolling median value (50th percentile) in a 0.4° × 0.4° box centered on the city center for the largest cities (New York City, Los Angeles, Chicago, Toronto, and Houston) and 0.2° × 0.2° box in all other cities.

In Figure 4, we demonstrate Method 3, in which we account for meteorology and chemical interactions using a chemical transport model. We create a theoretical TROPOMI column NO_2_ using ECCC's regional operational air quality forecast model (Moran et al., 2009; Pendlebury et al., 2018), which accounts for typical seasonal emission changes but not for any impacts due to the COVID‐19 lockdowns; this helps provide expected NO_2_ levels with a business as usual scenario. Around mid‐March there is often a divergence between the expected and observed NO_2_ in the major cities. Using this method, largest NO_2_ reductions due to COVID‐19 precautions are in Toronto, San Jose, and Las Vegas. Similar to Method 2, we find that NO_2_ changes are generally smaller in the Northeastern United States and Florida as compared to Method 1 after accounting for meteorology. In 15 of the 20 studied cities, we find that Methods 2 and 3, which utilize independent meteorological data sets, show similar biasing effects of meteorology (favorable vs. unfavorable) when compared to Method 1.

## 4 Conclusions and Discussion

We estimate that NO_2_ adjusted for seasonality and meteorology temporarily dropped between 9–43% in North American cities due to COVID‐19 precautions, with a median drop of 21.6% before and after COVID‐19 physical distancing. If the solar zenith angle is not accounted for, then the median NO_2_ drop is 39.9%; this represents the true change of NO_2_ in cities, but is not particularly helpful if parsing out emissions changes. Our reported median drop of 21.6% after accounting for meteorology is marginally lower than the 22.9% in a simple year‐to‐year comparison, which suggests that 2020 meteorology was slightly favorable for lower NO_2_, although these effects are most pronounced in the Northeastern United States and Florida.

Here we demonstrate two methodologies, Methods 2 and 3, to account for time‐varying effects of meteorology on NO_2_ concentrations. Although the NO_2_ concentration is also function of collocated chemical constituents, such as OH, VOCs, and O_3_, which also vary by year, the numbers reported here are closer to the true change in NO_*x*_ emissions than NO_2_ changes in the absence of accounting for meteorology. There are two main advantages for using Methods 2 and 3 to assess rapid changes in NO_*x*_ as compared to a year‐to‐year comparison of the same month or seasonal period. Year‐over‐year technological improvements in the North America are generally causing NO_*x*_ emissions to decrease over time (Goldberg, Lu, Oda, et al., 2019; Nopmongcol et al., 2017; Silvern et al., 2019; Zhang et al., 2018), although we find a statistically insignificant NO_2_ increase of 0.6% in our cities between 2019 and 2020 in the January–February average. Accounting for year‐over‐year changes would be more important if comparing 2020 values to years preceding 2019. There are two U.S. cities, Denver and Philadelphia, which have consistently lower values in 2020 than in 2019 preceding March, using Method 2. While it is conceivable that there may have been policies in these two cities that yielded significantly lower NO_*x*_ emissions in 2020 versus 2019 preceding March, Method 3 does not corroborate this and further, we do not think the evidence is strong enough to conclude this given the uncertainty related to meteorological conditions unaccounted for, such as snow cover differences between years.

A deficiency of our method is our reliance on a single satellite instrument and algorithm. It is known that the operational TROPOMI NO_2_ algorithm underestimates tropospheric vertical column NO_2_ in urban areas due to its reliance on a global model to provide shape profiles for the air mass factor (AMF); investigating the effects of the AMF bias on trends will be the subject of future work. Also, there may be a clear‐sky bias (Geddes et al., 2012) associated with TROPOMI retrievals, but the results presented here are generally consistent with studies using ground monitors over the coincident region (Bekbulat et al., 2020) and the reported CO_2_ emissions reductions due to COVID‐19 precautions (Le Quéré et al., 2020).

The estimates of NO_2_ changes using our methods appear to be reasonable given a quick bottom‐up emissions calculation. Initial statistics indicate vehicle miles traveled dropped by ~40% in April 2020 and that many industrial sources dropped modestly ~5–25% due to lockdown exemptions, NO_*x*_ reductions between 10% and 35% would be expected. San Jose, Los Angeles, and Toronto appear to have reductions at the high end of this range, while Miami, Minneapolis, and Dallas have values near the lowest end; further work will look into why these cities have reductions on the ends of the spectrum. Rapid assessments of NO_2_ changes—after normalized for seasonal and meteorological factors—can be used to highlight locations that may have had greater changes in activity and better understand the sources contributing to adverse air quality in each city.

## Supporting information

Supporting Information S1

## Data Availability

TROPOMI NO_2_ data can be freely downloaded from the European Space Agency Copernicus Open Access Hub or the NASA EarthData Portal (http://doi.org/10.5270/S5P-s4ljg54). The 100‐m wind and 2‐m surface temperatures from the ERA5 reanalysis can be freely downloaded from the Copernicus Climate Change (C3S) climate data store (CDS) (http://doi.org/10.24381/cds.adbb2d47) The intermediary data used to generate all figures can be found at http://doi.org/10.6084/m9.figshare.12721724. The submitted manuscript has been created by UChicago Argonne, LLC, Operator of Argonne National Laboratory (“Argonne”). Argonne, a U.S. Department of Energy Office of Science laboratory, is operated under Contract DE‐AC02‐06CH11357.
