# Supplementary material for: Disentangling the Impact of the COVID‐19 Lockdowns on Urban NO2 From Natural Variability
Source: Geophys Res Lett. 2020 Sep 5;47(17):e2020GL089269. doi: 10.1029/2020GL089269 (PMC7461033; doi:10.1029/2020GL089269)
Supplement: Supplementary file 1 — Supporting Information S1 [file GRL-47-0-s001.docx]

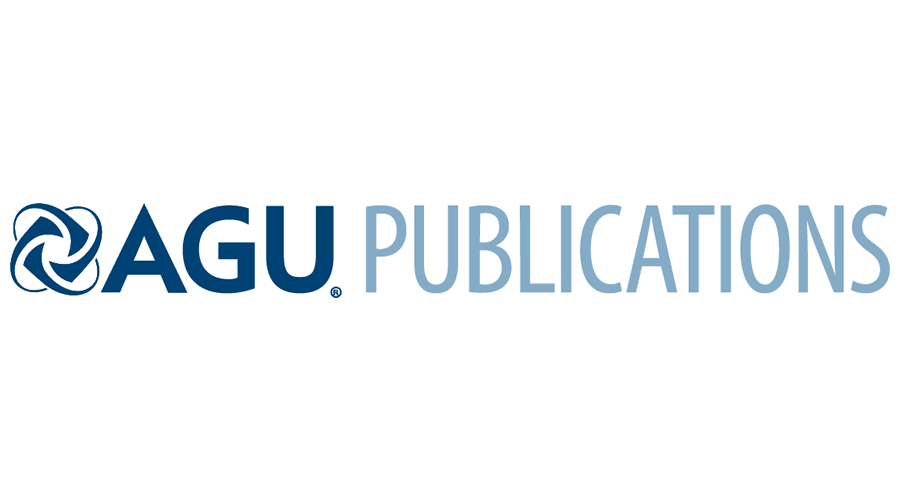


Supporting Information for:

**Disentangling the impact of the COVID-19 lockdowns on urban NO_2_ from natural variability**

Daniel L. Goldberg*^,1,3^, Susan C. Anenberg^1^, Debora Griffin^2^, Chris A. McLinden^2^, Zifeng Lu^3^, David G. Streets^3^

^1^Department of Environmental and Occupational Health, George Washington University, Washington, DC, U.S.

^2^Air Quality Research Division, Environment and Climate Change Canada (ECCC), Toronto, Ontario, Canada

^3^Energy Systems Division, Argonne National Laboratory, Lemont, IL, U.S.

^*^Corresponding author. Phone: (202)994-8102; Email: [dgoldberg@gwu.edu](mailto:dgoldberg@gwu.edu)

**This PDF file includes:**

Further description of TROPOMI NO_2_ processing technique and associated uncertainties

Further description of methodologies using to calculate NO_2_ drops during COVID-19

Figures S1 to S8

Table S1 to S2

1. TROPOMI NO_2_
   1. Air Mass Factors and Uncertainty Estimates

The slant tropospheric column is converted to a vertical column using a quantity known as the air mass factor (Palmer et al., 2001). The air mass factor is the most uncertain quantity in the retrieval algorithm (Lorente et al., 2017), and is a function of the surface reflectance, the NO_2_ vertical profile, and scattering in the atmosphere among other factors (Lamsal et al., 2014). Using accurate and high-resolution data (spatially and temporally) as inputs in calculating the air mass factor can significantly reduce the overall errors of the air mass factor (Choi et al., 2019; Goldberg et al., 2017; Laughner et al., 2016, 2019; Lin et al., 2015; Liu et al., 2019; Russell et al., 2011; Zhao et al., 2020) and thus the tropospheric vertical column content.

Operationally, the TM5-MP model (1 × 1° resolution) (Williams et al., 2017) is used to provide the NO_2_ vertical shape profile and the climatological Lambertian Equivalent Reflectivity (0.5 × 0.5° resolution) (Kleipool et al., 2008) is used to provide the surface reflectivities. The operational air mass factor calculation does not explicitly account for aerosol absorption effects, which are accounted for in the effective cloud radiance fraction. While the operational product does have larger uncertainties in the tropospheric column contents than a product with higher spatial resolution inputs, we limit our analysis to relative trends, which dramatically reduces this uncertainty. ﻿The uncertainty in any daily measurement in the operational slant column data has been assigned to be approximately 5.7 × 10^14^ molecules-cm^-2^ (van Geffen et al., 2020). This equates to roughly a 5-10% uncertainty over polluted areas. However, because we are averaging over many days (~20-40), we assume that random errors will cancel due to the large number of observations used. This leaves only the systematic errors. Here, we assign the AMFs and tropospheric vertical column contents a systematic uncertainty of 20% in the trends (McLinden et al., 2014). This systematic uncertainty may be largest over areas with changing snow cover, such as Minneapolis, Chicago, Toronto, and Montreal. We calculate total uncertainty as the quadrature of the uncertainty associated with this potential systematic bias and the standard deviation of the three Methods. These are listed in Table S2.

**1.2 Re-gridding of TROPOMI NO_2_**

For our analysis we re-grid the operational TROPOMI tropospheric vertical column NO_2_, with native pixels of approximately 3.5 × 7 km^2^, to a newly defined 0.01° × 0.01° grid (approximately 1 × 1 km^2^) centered over the continental United States (CONUS; corner points: SW: 24.5° N, 124.75° W; NE: 49.5° N, 66.75° W). Before re-gridding, the data are filtered so as to use only the highest quality measurements (quality assurance flag (QA_flag) > 0.75).

1. **Description of Methodologies 2 & 3**

**2.1 Method 2: Normalization of Daily TROPOMI NO_2_ using ERA5**

We use TROPOMI NO_2_ data from 2018 – 2019 as analog data to normalize 2020 data. Essentially, our method is searching through the 2018 – 2019 archive to find a meteorological analog to the current conditions and then adjusting the current day’s conditions based off that analog.

For each day of the record, we modify the original observed TROPOMI NO_2_ based on its value compared to a "baseline" which we set as a weekday in April with 3 m/s southwest winds. For each day, *n*, and each city, *i*, the normalized NO_2_, $\hat{{NO}_{2}}$ , is calculated as follows:

$${\hat{{NO}_{2}}}_{n,i}=\frac{{{NO}_{2}}_{n,i}}{{f_{total}}_{n,i}}$$

The subscript *i* represents a city-specific average within a 0.4° × 0.4° box surrounding the city center.

The four adjustment factors are: sun angle, wind speed, wind-direction, and day-of-week. While other conditions affect NO_2_ amounts they are either interrelated to the aforementioned factors or can be considered secondary. The wind-speed and wind-direction factors are computed separately for each city. Each of the four individual factors are multiplied together to get a "total adjustment factor". The “total adjustment factor”, *f_total_* is calculated for each day, *n*, and each city, *i*, as follows:

$${f_{total}}_{n,i}=\left[ f_{sun-angle} \right]_{n}\left[ f_{day-of-week} \right]_{n}\left[ f_{wind-speed} \right]_{n,i}\left[ f_{wind-dir} \right]_{n,i}$$

For the sun angle factor, we calculate this using a cosine fit, which is based on the climatological seasonality of NO_2_ which peaks in winter months, and lowers during summer months (Goldberg et al., n.d.; Ialongo et al., 2016; Shah et al., 2020); an illustrative example is shown in Figure S8. For each Julian date, *n*, the sun angle factor (*f_sun-angle_*) can be calculated as follows:

$${f_{sun-angle}}_{n}=\frac{0.75+0.25*cos\left[ 2\pi\frac{n+11}{365} \right]}{0.75+0.25*cos\left[ 2\pi\frac{n_{d}+11}{365} \right]}$$

At the winter solstice, December 21^st^ (n = -11 or n = 354) the numerator value is 1 and at the summer solstice, June 21^st^ (n = 171) the numerator value is 0.5. The variable n_d_ represents the normalization day, in this case April 15^th^ (n_d_ = 105). The aforementioned equation is only valid for locations north of the Tropic of Cancer (23.4°N).

For the wind speed factor, we fit a third-order polynomial using analog winds speeds from the 2018 – 2019 TROPOMI time frame. Wind speeds of 5 m/s would yield a correction factor of 1. Values larger than 1 represent winds slower than 5 m/s and values smaller than 1 represent winds faster than 5 m/s. This fit allows us to calculate a correction factor given any city-specific wind speed.

For the wind direction factor, we calculate a correction factor normalized to southwest winds. Wind directions are grouped into the following categories: 0 – 90 º are southwest, 90 – 180 º are northwest, 180 – 270 º are northeast, and 270 – 360 º are southeast. Days with southwest winds yield a correction factor of 1.

Lastly, for the day-of-week factor, we assume 15% lower values on Saturdays and 30% lower values on Sundays. We assume all weekdays have similar emissions rates to each other. Weekdays have a factor of 1, Saturdays a factor of 0.85 and Sundays a factor of 0.70. These assumptions are broadly consistent with literature demonstrating day-of-week NO_X_ emissions patterns (Beirle et al., 2003; Goldberg et al., n.d.; Russell et al., 2010; Stavrakou et al., 2020).

As an example, a stagnant day in January may be lowered by a factor of ~2 to "normalize" to a 5 m/s April weekday, whereas a very windy weekend day in April might be increased by a factor of 1.5 to account for the faster than normal winds and the weekend effect.

**Method 3: Normalization of Daily TROPOMI NO_2_ using a CTM**

We infer expected NO_2_ columns (V_ex_) during the lock-down period (t_covid_) using the output from the GEM-MACH model (Moran et al., 2009; Pendlebury et al., 2018). The operational version of the model, used in this study, has a 10 × 10 km^2^ grid cell size with 80 vertical levels (from the surface to about 0.1 hPa), provides hourly output, and includes emissions, chemistry, dispersion, and removal processes of 41 gaseous and eight particle species. The emissions used in the model are processed using the Sparse Matrix Operator Kernel Emissions (SMOKE)(Coats, n.d.) and account for daily, weekly, and seasonal cycles; changes in emissions due to the COVID-10 lock-downs are not considered in the model framework. ﻿Anthropogenic emissions in the GEM-MACH model are based on a projected 2017 U.S. National Emission Inventory (NEI) from the U.S. EPA's 2011 policy platform (https://www.epa.gov/air-emissions-modeling/2011-version-63-platform); Canadian emissions are based on the 2013 Canadian Air Pollutant Emission Inventory (APEI) (https://www.canada.ca/en/environment-climate-change/services/pollutants/air-emissions-inventory-overview.html); Mexican emissions are based on the 2008 Mexican NEI provided as part of the U.S. EPA's 2011 policy platform. Biogenic emissions in the model are calculated on-line based on daily meteorology using the Biogenic Emissions Inventory System (BEIS).

In a first step the GEM-MACH NO_2_ vertical levels in the boundary layer (up to approximately 2 km) are summed to a column amount using the model’s pressure and temperature profile(Côté et al., 1998). Since the GEM-MACH model currently does not contain any NO_x_ sources in the free troposphere (such as aircraft or lightning emissions), the NO_2_ model concentrations decrease to 0 above the planetary boundary layer (PBL). A free tropospheric column (from 2 km to 12 km) is added to the GEM-MACH PBL vertical column densities (VCDs) using a monthly GEOS-Chem run (0.5x0.67⁰ resolution, version v8-03-01; <http://www.geos-chem.org>)(Bey et al., 2001; McLinden et al., 2014). The model VCDs are then mapped in space and time to the TROPOMI observations. For every valid TROPOMI observation (QA value > 0.75), we summon the GEM-MACH NO_2_ VCD corresponding to the exact same time and space. We only use GEM-MACH data when there is a valid TROPOMI pixel and discard the rest of the model data (it is never used for this analysis). The TROPOMI and GEM-MACH data are then averaged over the city center using a 28-day running mean.

The expected VCDs (V_ex_) are the 28-day running means of the modelled VCDs (V_M_) during the lockdown period (t_covid_). V_ex_ is scaled to remove any bias between the model and satellite (V_T­_) for the pre-lockdown period (t_pre_, between February 1^st^ and March 1^st^ 2020):

$$V_{ex}\left( t_{covid} \right)=V_{M}\left( t_{covid} \right)\cdot mean\left( \frac{V_{T}\left( t_{pre} \right)}{V_{M}\left( t_{pre} \right)} \right).$$

Depending on the city, some dates within the t_pre_ time period may not be considered for the scaling, if there is a strong divergence between the model and the observations.

The estimated NO_2_ drop is the average of the difference between the expected VCDs, V_ex_(t_covid_), and the observed TROPOMI VCDs, V_T_(t_covid_), between March 28^th^ and April 16^th^, 2020 using the daily 28-day running means as shown in Figure 4.

1. Supplemental Figures


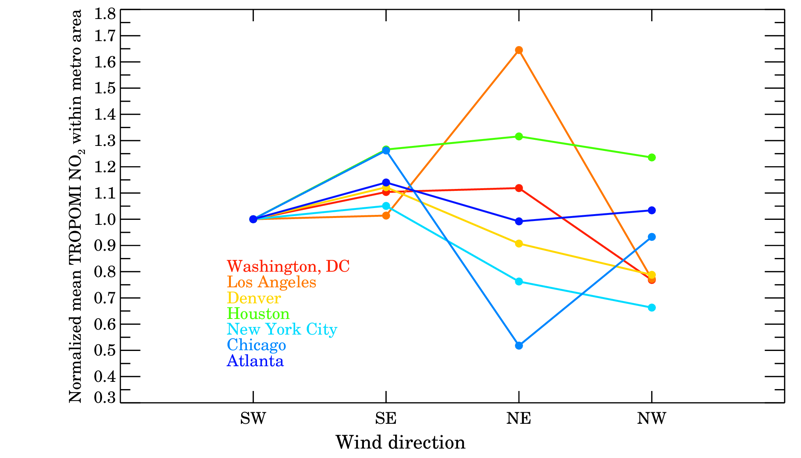

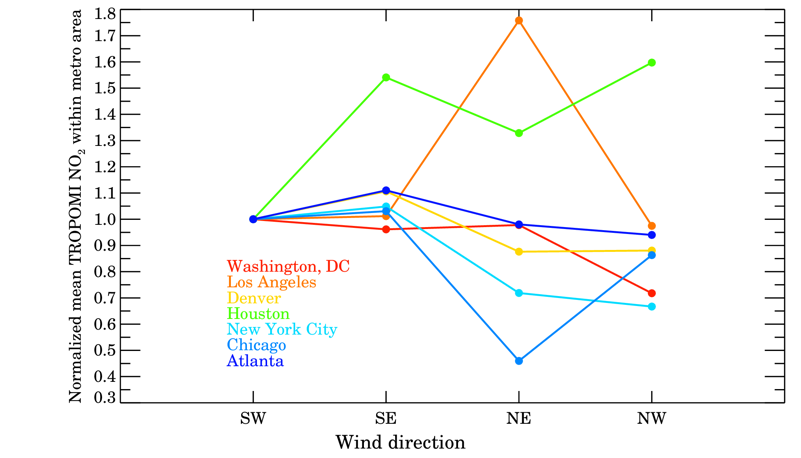


Moderate winds only

All winds

**Figure S1.** Variations as a function of wind direction for seven cities normalized to southwesterly winds, using (left) all wind speeds (same as Figure 1i) and (right) only moderate (4-6 m/s) wind speeds.


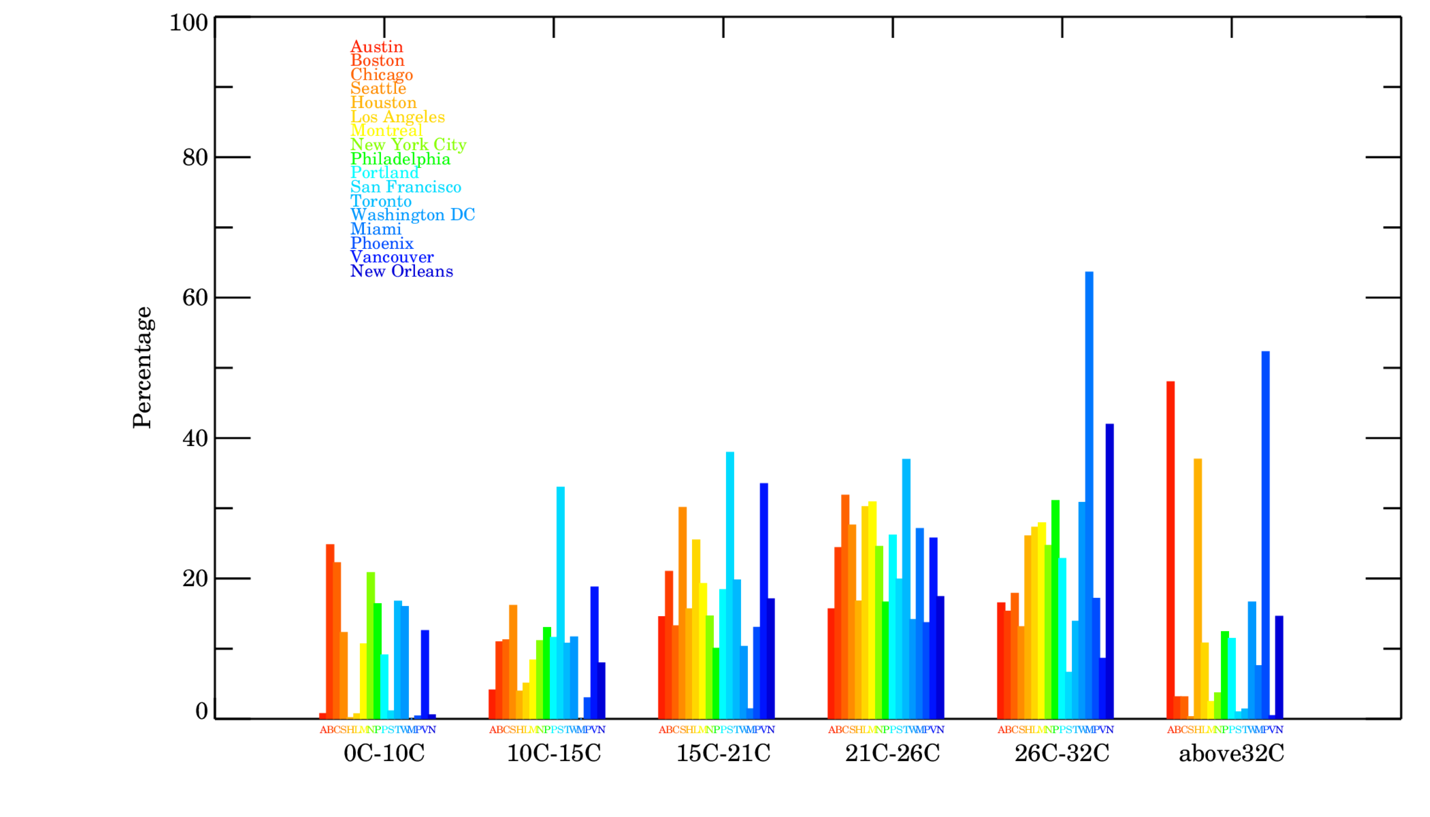


**Figure S2**. Frequency of daily maximum 2-m temperature within each bin, according to the ERA5 re-analysis. Each bar is a different city as noted by list in top left. These climatologies are calculated during the TROPOMI record preceding Jan 1, 2020 (May 1, 2018 – Dec 31, 2019).


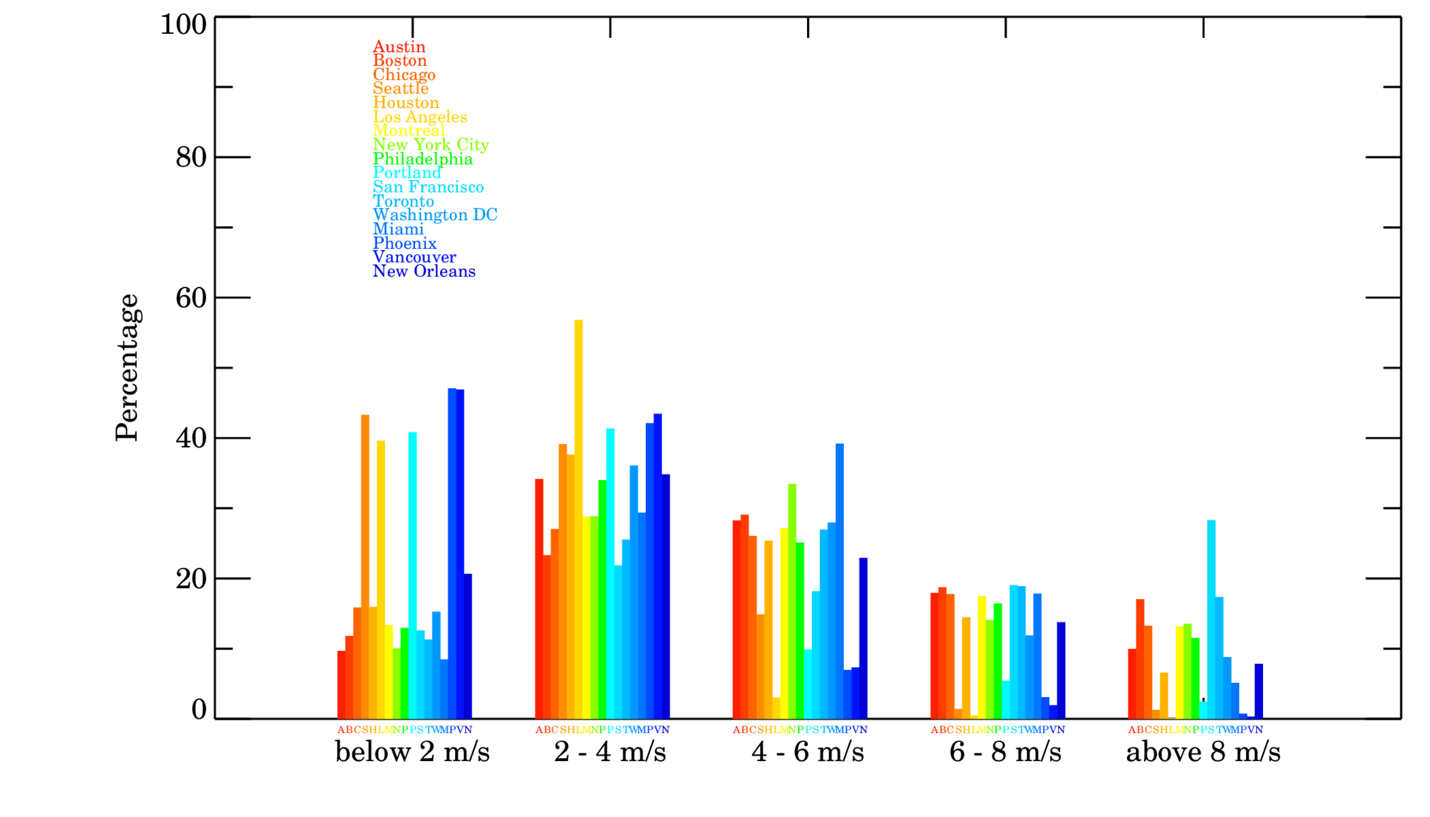


**Figure S3**. Frequency of 100-m afternoon (16Z-21Z) wind speed within each bin, according to the ERA5 re-analysis. Each bar is a different city as noted by list in top left. These climatologies are calculated during May 1, 2018 – Dec 31, 2019.


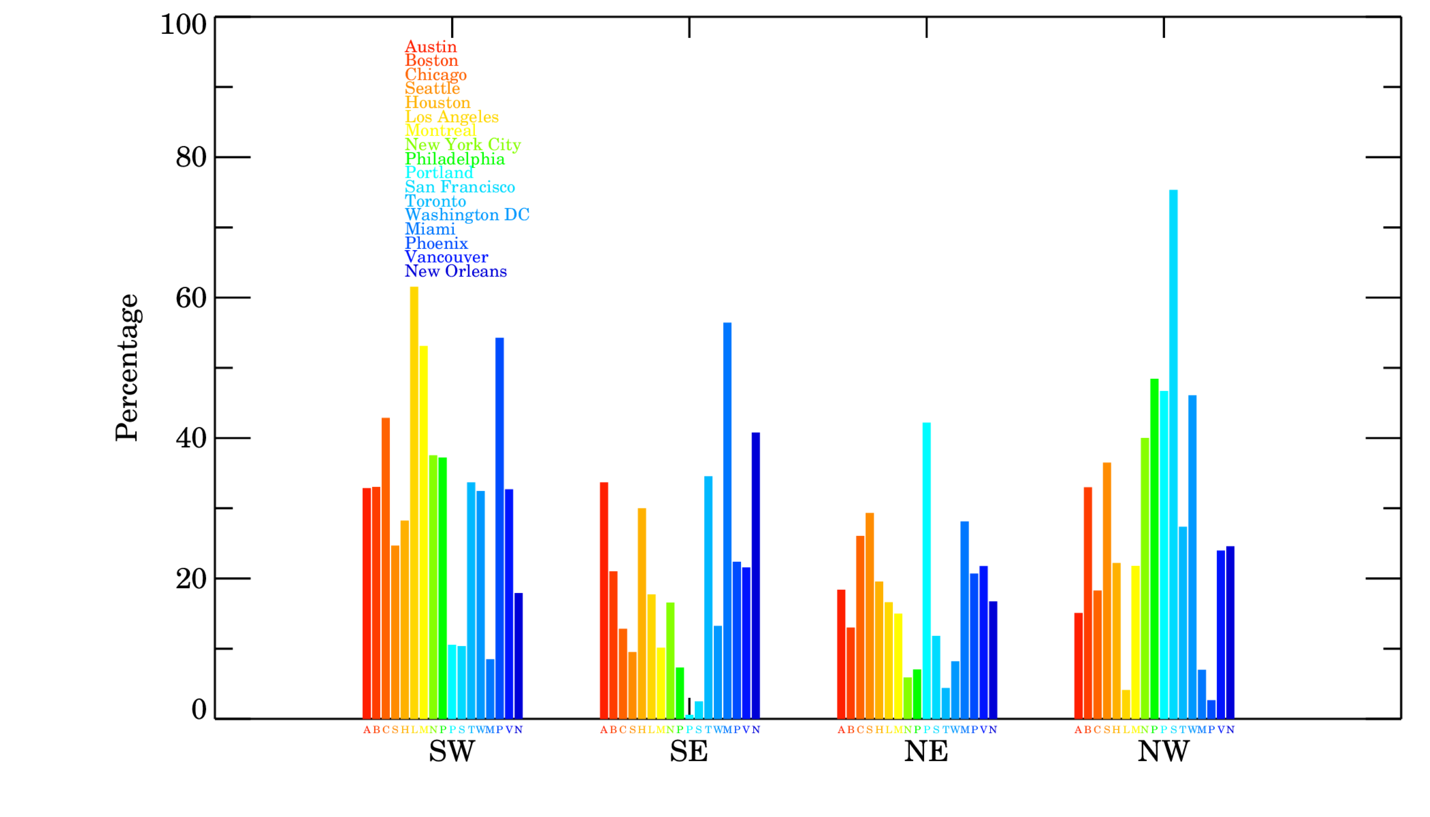


**Figure S4**. Frequency of 100-m afternoon (16Z-21Z) wind direction within each bin, according to the ERA5 re-analysis. Each bar is a different city as noted by list in top left. These climatologies are calculated during May 1, 2018 – Dec 31, 2019.


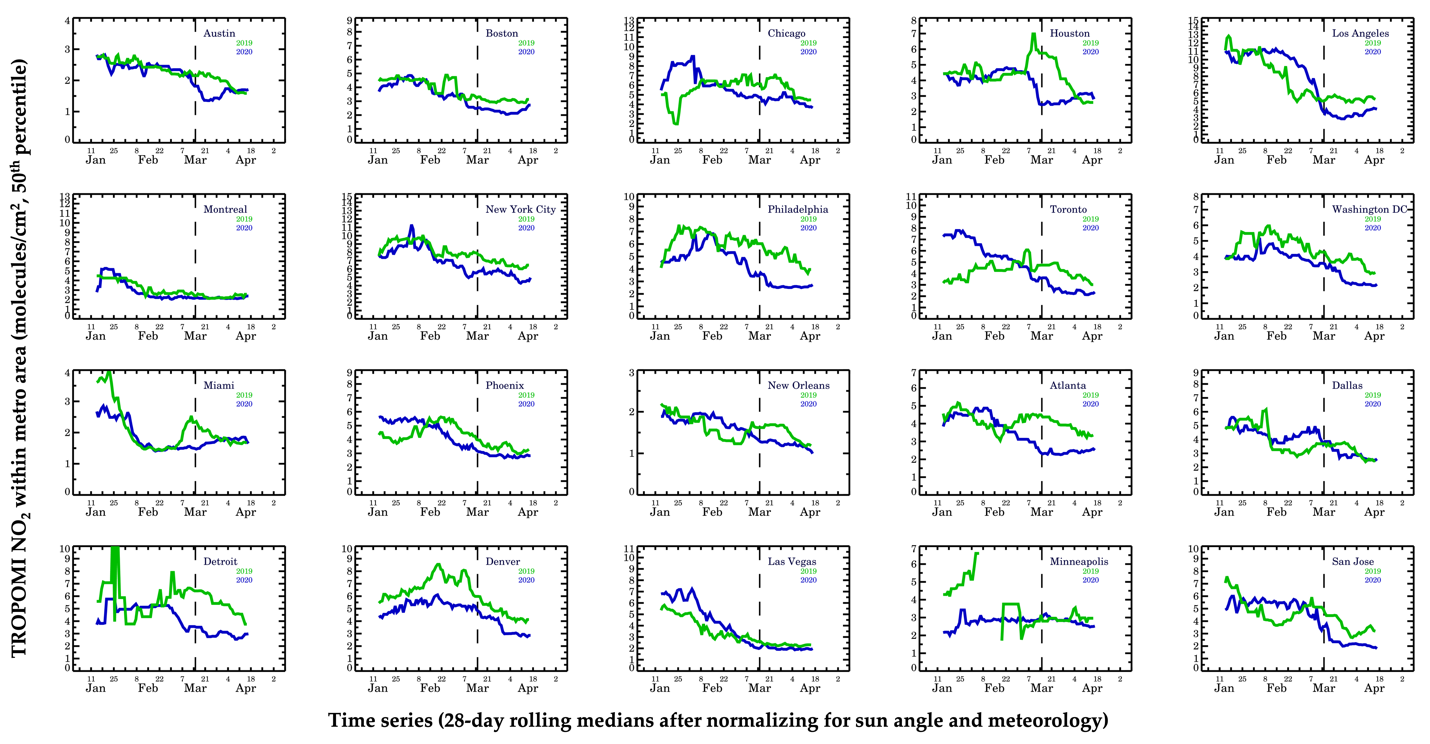


**Figure S5**. Trends in TROPOMI NO_2_ since January 1 in 2019 and 2020. The lines represent the 28-day rolling median value (50^th^ percentile) in a 0.4° × 0.4° box centered on the city center for the largest cities (New York City, Los Angeles, Chicago, Toronto, Houston) and 0.2° × 0.2° box in all other cities.


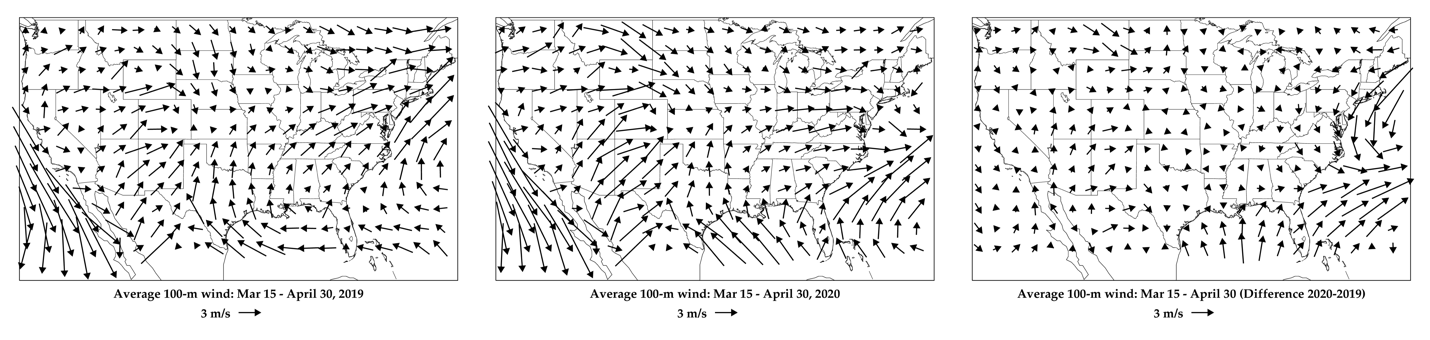


**Figure S6**. Average 100-m afternoon (16Z-21Z) wind speed and direction for March 15 – April 30 in (left) 2019, (center) 2020, (right) difference between the two years, according to the ERA5 re-analysis.


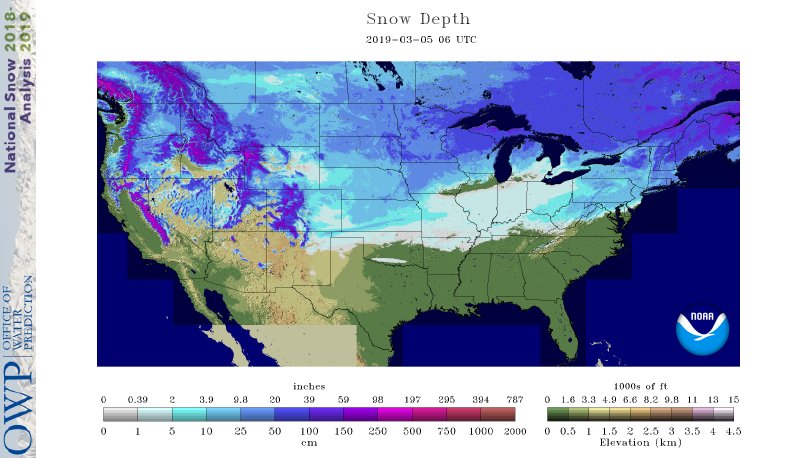

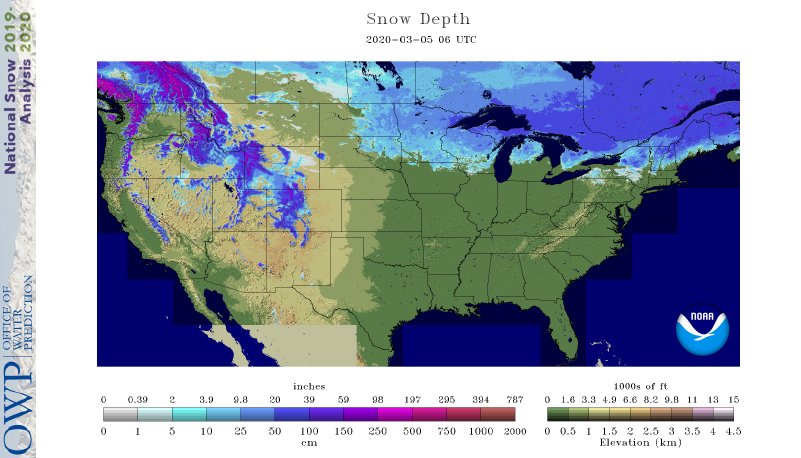


**Figure S7**. Snow cover extent and snow depth on March 5, 2019 vs. March 5, 2020. CONUS covered by snow: 57.1% in 2019 vs. 19.8% in 2020. Images and data acquired from: <https://www.nohrsc.noaa.gov/nsa/>


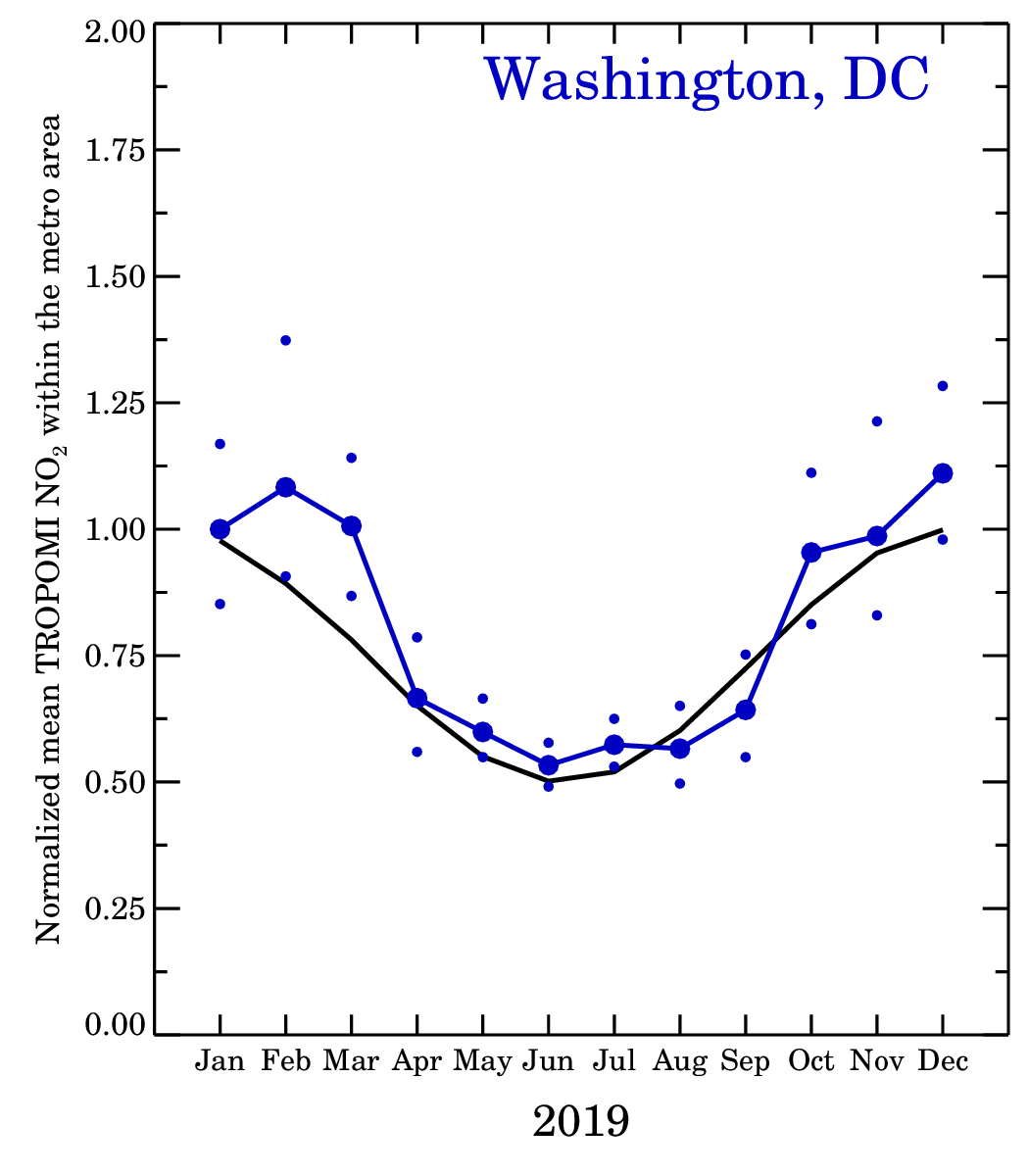


**Figure S8.** Cosine fit of the normalized monthly TROPOMI NO_2_ using data for Washington, D.C. as an illustrative example.

1. **Supplemental Tables**

**Table S1. Latitude, longitude, and box width size of each of the 20 cities**

**Table S2.** Uncertainties associated with our methodology. Uncertainties are calculated as the quadrature of any potential systematic bias (20%) and the standard deviation of Methods 1 – 3.


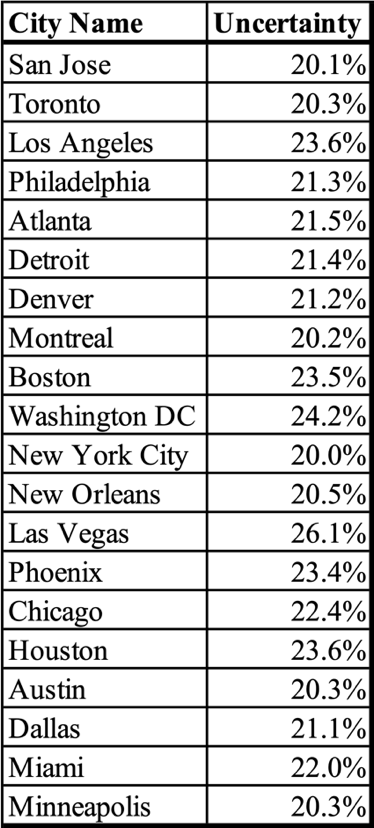


**References**

Beirle, S., Platt, U., Wenig, M., & Wagner, T. (2003). Weekly cycle of NO2 by GOME measurements: A signature of anthropogenic sources. *Atmospheric Chemistry and Physics*, *3*(6), 2225–2232. https://doi.org/10.5194/acp-3-2225-2003

Bey, I., Jacob, D. J., Yantosca, R. M., Logan, J. A., Field, B. D., Fiore, A. M., et al. (2001). Global modeling of tropospheric chemistry with assimilated meteorology: Model description and evaluation. *Journal of Geophysical Research: Atmospheres*, *106*(D19), 23073–23095. https://doi.org/10.1029/2001JD000807

Choi, S., Lamsal, L. N., Follette-Cook, M., Joiner, J., Krotkov, N. A., Swartz, W. H., et al. (2019). Assessment of NO2 observations during DISCOVER-AQ and KORUS-AQ field campaigns. *AMTD*. https://doi.org/10.5194/amt-2019-338

Coats, C. J. (n.d.). *High-performance algorithms in the Sparse Matrix Operator Kernel Emissions (SMOKE) Modeling System, American Meteorological Society, Atlanta, GA. USA, proceedings of the Ninth AMS Joint Conference on Applications of Air Pollution Meteorology with AWMA.*

Côté, J., Gravel, S., Méthot, A., Patoine, A., Roch, M., & Staniforth, A. (1998). The Operational CMC–MRB Global Environmental Multiscale (GEM) Model. Part I: Design Considerations and Formulation. *Monthly Weather Review*, *126*(6), 1373–1395. https://doi.org/10.1175/1520-0493(1998)126<1373:TOCMGE>2.0.CO;2

van Geffen, J., Boersma, K. F., Eskes, H., Sneep, M., ter Linden, M., Zara, M., & Veefkind, J. P. (2020). S5P TROPOMI NO&amp;lt;sub&amp;gt;2&amp;lt;/sub&amp;gt; slant column retrieval: method, stability, uncertainties and comparisons with OMI. *Atmospheric Measurement Techniques*, *13*(3), 1315–1335. https://doi.org/10.5194/amt-13-1315-2020

Goldberg, D. L., Anenberg, S. C., Mohegh, A., Lu, Z., & Streets, D. G. (n.d.). TROPOMI NO2 in the United States: A detailed look at the annual averages, weekly cycles, effects of temperature, and correlation with PM2.5. *Pre-Print*.

Goldberg, D. L., Lamsal, L. N., Loughner, C. P., Swartz, W. H., Lu, Z., & Streets, D. G. (2017). A high-resolution and observationally constrained OMI NO2 satellite retrieval. *Atmospheric Chemistry and Physics*, *17*(18), 11403–11421. https://doi.org/10.5194/acp-17-11403-2017

Ialongo, I., Herman, J. R., Krotkov, N., Lamsal, L. N., Folkert Boersma, K., Hovila, J., & Tamminen, J. (2016). Comparison of OMI NO2 observations and their seasonal and weekly cycles with ground-based measurements in Helsinki. *Atmospheric Measurement Techniques*, *9*(10), 5203–5212. https://doi.org/10.5194/amt-9-5203-2016

Kleipool, Q. L., Dobber, M. R., de Haan, J. F., & Levelt, P. F. (2008). Earth surface reflectance climatology from 3 years of OMI data. *Journal of Geophysical Research Atmospheres*, *113*(18), 1–22. https://doi.org/10.1029/2008JD010290

Lamsal, L. N., Krotkov, N. A., Celarier, E. A., Swartz, W. H., Pickering, K. E., Bucsela, E. J., et al. (2014). Evaluation of OMI operational standard NO2 column retrievals using in situ and surface-based NO2 observations. *Atmospheric Chemistry and Physics*, *14*(21), 11587–11609. https://doi.org/10.5194/acp-14-11587-2014

Laughner, J. L., Zare, A., & Cohen, R. C. (2016). Effects of daily meteorology on the interpretation of space-based remote sensing of NO2. *Atmospheric Chemistry and Physics*, *16*(23), 15247–15264. https://doi.org/10.5194/acp-16-15247-2016

Laughner, J. L., Zhu, Q., & Cohen, R. C. (2019). Evaluation of version 3.0B of the BEHR OMI NO2 product. *Atmospheric Measurement Techniques*, *12*(1), 129–146. https://doi.org/10.5194/amt-12-129-2019

Lin, J. T., Liu, M. Y., Xin, J. Y., Boersma, K. F., Spurr, R., Martin, R. V., & Zhang, Q. (2015). Influence of aerosols and surface reflectance on satellite NO2 retrieval: Seasonal and spatial characteristics and implications for NOx emission constraints. *Atmospheric Chemistry and Physics*, *15*(19), 11217–11241. https://doi.org/10.5194/acp-15-11217-2015

Liu, M., Lin, J., Boersma, K. F., Pinardi, G., Wang, Y., Chimot, J., et al. (2019). Improved aerosol correction for OMI tropospheric NO2 retrieval over East Asia: constraint from CALIOP aerosol vertical profile. *Atmospheric Measurement Techniques*, *12*(1), 1–21. https://doi.org/10.5194/amt-12-1-2019

Lorente, A., Folkert Boersma, K., Yu, H., Dörner, S., Hilboll, A., Richter, A., et al. (2017). Structural uncertainty in air mass factor calculation for NO2 and HCHO satellite retrievals. *Atmospheric Measurement Techniques*, *10*(3), 759–782. https://doi.org/10.5194/amt-10-759-2017

McLinden, C. A., Fioletov, V. E., Boersma, K. F., Kharol, S. K., Krotkov, N., Lamsal, L. N., et al. (2014). Improved satellite retrievals of NO2 and SO2 over the Canadian oil sands and comparisons with surface measurements. *Atmospheric Chemistry and Physics*, *14*(7), 3637–3656. https://doi.org/10.5194/acp-14-3637-2014

Moran, M. D., Ménard, S., Talbot, D., Huang, P., Makar, P. A., Gong, W., et al. (2009). *Particulate-Matter Forecasting with GEM-MACH15, A New Canadian Air-Quality Forecast Model. In Air Pollution Modeling and Its Application XX*. Retrieved from http://www.nato.int/science

Palmer, P. I., Jacob, D. J., Chance, K., Martin, R. V., Spurr, R. J. D., Kurosu, T. P., et al. (2001). Air mass factor formulation for spectroscopic measurements from satellites: Application to formaldehyde retrievals from the Global Ozone Monitoring Experiment. *Journal of Geophysical Research: Atmospheres*, *106*(D13), 14539–14550. https://doi.org/10.1029/2000JD900772

Pendlebury, D., Gravel, S., Moran, M. D., & Lupu, A. (2018). Impact of chemical lateral boundary conditions in a regional air quality forecast model on surface ozone predictions during stratospheric intrusions. *Atmospheric Environment*, *174*, 148–170. https://doi.org/10.1016/j.atmosenv.2017.10.052

Russell, A. R., Valin, L. C., Bucsela, E. J., Wenig, M. O., & Cohen, R. C. (2010). Space-based constraints on spatial and temporal patterns of NOx emissions in California, 2005-2008. *Environmental Science and Technology*, *44*(9), 3608–3615. https://doi.org/10.1021/es903451j

Russell, A. R., Perring, A. E., Valin, L. C., Bucsela, E. J., Browne, E. C., Wooldridge, P. J., & Cohen, R. C. (2011). A high spatial resolution retrieval of NO2 column densities from OMI: Method and evaluation. *Atmospheric Chemistry and Physics*, *11*(16), 8543–8554. https://doi.org/10.5194/acp-11-8543-2011

Shah, V., Jacob, D. J., Li, K., Silvern, R. F., Zhai, S., Liu, M., et al. (2020). Effect of changing NOx lifetime on the seasonality and long-term trends of satellite-observed tropospheric NO2 columns over China. *Atmospheric Chemistry and Physics Discussions*, *20*(3), 1483–1495. https://doi.org/10.5194/acp-2019-670

Stavrakou, T., Müller, J.-F., Bauwens, M., Boersma, K. F., & van Geffen, J. (2020). Satellite evidence for changes in the NO2 weekly cycle over large cities. *Scientific Reports*, *10*(1), 10066. https://doi.org/10.1038/s41598-020-66891-0

Williams, J. E., Folkert Boersma, K., Le Sager, P., & Verstraeten, W. W. (2017). The high-resolution version of TM5-MP for optimized satellite retrievals: Description and validation. *Geoscientific Model Development*, *10*(2), 721–750. https://doi.org/10.5194/gmd-10-721-2017

Zhao, X., Griffin, D., Fioletov, V., McLinden, C. A., Cede, A., Tiefengraber, M., et al. (2020). Assessment of the quality of TROPOMI high-spatial-resolution NO2 data products in the Greater Toronto Area. *Atmospheric Measurement Techniques*, *13*(4), 2131–2159. https://doi.org/10.5194/amt-13-2131-2020
